# Renoprotective effect of hydroalcoholic extract of *Rheum ribes* root in diabetic female rats

**Published:** 2014

**Authors:** Shokri Hamzeh, Farah Farokhi, Reza Heydari, Ramin Manaffar

**Affiliations:** 1 *Department of Biology, Faculty of Basic Science, Urmia University, Urmia, I. R. Iran*; 2 *Artemia and Aquatic Animals Research Institute of Urmia University, Urmia, I. R. Iran*

**Keywords:** *Alloxan*, *Diabetes mellitus*, *Rheum ribes root extract*

## Abstract

**Objective:** Medical plants, as rich sources of natural antioxidants with antidiabetic effects, are used worldwide to diminish a variety of symptoms and many diseases. *R. ribes *L., which belongs to the family of polygonaceae, can provide symptomatic relief and assist in the prevention of the secondary complications of the diabetes.

**Material and Methods:** 36 female adult rats were randomly divided into 6 groups of 6. Normal Control groups treated with normal saline. Positive control groups treated with hydroalcohlic extract of *R. ribes* root (150 mg/kg) daily by gavages for 4 consecutive weeks. Diabetes was inducedby injection of 120 mg/kg alloxan monohydrate intraperitoneally. Two diabetic groups were treated with different doses of *R. ribes* root extract. The sixth diabetic groups were treated with glibenclamide (0.6 mg/kg). At the end of 28 days, blood samples were collected and their kidney tissues were processed for light microscopy.

**Results:** The results showed that hydro-alcoholic extract of *R. ribes *decreased the level of glucose, cholesterol, triglyceride, urea and creatinine in diabetic rats (p<0.05) in compared with diabetic rats, while the level of HDL increased at the same group (p<0.05). Histopathological changes of kidney samples were comparable with respective control. In diabetic rats, kidney sections showed atrophy of glomerular capillaries with increased Bowman's space and acute tubular necrosis. The groups that were treated with *R. ribes* root were improved towards normal condition.

**Conclusion:** It is interesting to note that hydroalcohlic extract of *R. ribes *root improves renal dysfunction in alloxan-induced diabetic rats through controlling blood glucose and renal protective effects.

## Introduction

Diabetes is characterized by chronic hyperglycemiawith disturbances of carbohydrate, fat and protein metabolism. The number of adults affected by diabetes in the world is expected to increase from 135 million in 1995 to 300 million in the year 2025 (Liu et al., 2007 ▶). The majority of this diabetic population will emerge from developing countries (Shaw et al., 2010 ▶). 

In type 1 diabetes β-cell destruction usually leads to absolute insulin deficiency and patients are reliant upon receiving insulin regularly (Ranjan and Raman jam, 2002 ▶). Type 2 diabetes is a heterogeneous type, a combination of insulin resistance and insulin deficiency (Lokesh and Amit, 2006 ▶). Insulin resistance, which has been attributedto elevated levels of free fatty acids in plasma, (Halder et al., 2003 ▶) leads to decreased glucose transport into muscle cells, elevated hepatic glucose production, and increased breakdown of fat. The aim of therapy in diabetes is to achieve normoglycemia to prevent later micro- vascular complications (retinopathy, nephropathy and microangiopathy). Intensive therapy to achieve glycemic control has been shown to significantly diminish the risk of long-term complications (DCCT, 2002 ▶).

Medicinal plants have always been an important source for finding new remedies for human health problems. Traditionally, numerous herbs have been recommended for treatment of diabetes. Also, antidiabetic effects of so many plants have been reported by many researchers (Ghorbani , 2013 ▶). According to the literature, more than 800 plants are discovered to have antidiabetic properties (Eddouks and Maghrani, 2004 ▶). Some of these traditional remedies have been studied experimentally (Marles and Farnsworth, 1996 ▶). Some herbs have been proven to help in the regeneration of ß-cells and in overcoming resistance. In addition, some others have antioxidant activity and cholesterol-lowering properties which can be very useful in medicine. The restrictions of available oral anti-diabetic medicines in efficacy or safety have encouraged researchers to discover new drugs with less side effects. Most of the plants contain glycosides, alkaloids, terpenoids, flavonoids, carotenoids, etc., that are frequently implicated as having antidiabetic effect (Malviya et al., 2010 ▶). *R. ribes *L. belongs to the family of polygonaceae. It’s used for medicinal purposes; also its fresh stems and the petioles are consumed as a vegetable. It is commonly found in eastern Turkey, Lebanon and Iran (Krishnaiah et al., 2010 ▶). *R. ribes* has been used in the treatment of different symptoms like laxative, antidiarrhoea, gastrointestinal hemorrhage and treatment injuries (Tyler et al., 1988 ▶; Machlean et al., 2001 ▶). The renal failure, glucose uptake modification and diabetic nephropathy in human,rat and rats respectively were described by budavari and Li-Is (Budavari, 1989 ▶; Li-Is, 1993 ▶). The decoction extract of *R. ribes* roots possess significant blood sugar lowering activity in alloxan-induced diabetic rat, although this extract did not show hypoglycemic action in healthy rats (Özbeket al., 2004 ▶). Hypocholesterolemic effects of both ethanolic and aqueous extracts of *R. ribes *in rabbit have been also reported (Hadjzadeh et al., 2004 ▶). The roots of *R.ribes*, collected from Bingöl, contain tannins (8%) and anthracene derivatives (0.025%) (Baytop, 1999 ▶). According to the results of the chemical study on material collected from the plant substances like Erzincan, chrysophanol, physcion, Rhein, aloe-emodin, physcion-8-O-glucoside, aloe-emodin-8-Oglucoside, sennoside A and rhaponticin were found in the subterranean parts of the plant (Tuzlacıetal., 1991 ▶). Considering the phenolic constituent profile of *R. ribes*, particularly their flavonoids, stilbenes and anthraquinones, they appear to provide a potential source of antioxidants (Matsuda et al., 2001 ▶). According to the best of our knowledge, there is not a systematic studyon the hypoglycemic effect of *R. ribes*. The present study aimed to investigate the effects of hydroalcoholic extract of* R. ribes* root on diabetic Rats. The results of experiment were compared with the results of glibenclamide, a standard hypoglycemic agent.

## Materials and Methods

**Materials preparation**

Alloxan monohydrate and chloroform were purchased from Sigma Chemicals, Germany. The glibenclamide was obtained from Minoo Company, Iran. Fresh, green *R. ribes* plants were collected from *the Dalavan* Mountains of West Azerbaijan in northwest of Iran in frontier localities between Iran and Iraqin May 2010. The species of the plants were authenticated by Department of Biology, science department, Urmia University. The collected plants were cut into small pieces before drying. Powdered plant materials (50 gr) were extracted with ethanol (300 ml) by Sohxlet apparatus during a12 h period. After filtration, the solvent was removed under reduced pressure using a Rotavapor-RE to give a concentrated extract. The residue subsequently was stored in -20° C for the next operation.

**Diabetes induction**

36 female Wistar rats with BW of 200±20 g were purchased from Pasteur Institute, Iran, and were kept in animal houses of Uremia University. They were kept at 20±5°C, relative humidity of 30±5%, and light/dark cycle for 12h. The animals were allowed free access to tap water and standard laboratory rat food. All experimental procedures involving animals were approved by the Animal Research Ethics Committee of Urmia University, Faculty of Sciences, Uremia, Iran.

All Rats were passed 7days of adaptation in to the cages condition. Subsequently they were fasted overnight and 120 mg/kg of alloxan monohydrate freshly dissolved in normal saline was injected intra-peritoneal (Huang et al., 2006 ▶). After finishing the alloxan treatment, all animals were exposed to free food and water. A blood glucose test measurement was performed 2 days after alloxan injection and used as parameters to obtain matching pairs of rats with diabetes of similar level of severity (Jothivel et al., 2007 ▶).

**Experimental design**

The animals were divided into six group so fsix rats per group. In this experiment first group was control treated with normal saline (NC), second group as positive control (normal control) treated with extract of root *R. ribes *150 mg/kg body weight (N+RR150), third group as diabetic received normal saline rats (DC), forth group as diabetic rats treated with extract of *R. ribes* root (75 mg/kg body weight) (DC+RR75), fifth group was diabetic rats treated with extract of *R. ribes* root (150mg/kg body weight) (DC+RR150), and the final group was the diabetic rats treated with glibenclamide (0.6 mg/kg of body weight), (DC+G) (Kazemi et al.,2010 ▶). At the end of experiment (In the 28 day), the animals were fasted up to 12 hours and weighed and anesthetized with chloroform (Pharmaceutical Partners of Japan). Blood samples were collected from the animals' heart and the serum was separated by centrifugation (3000 rpm at 4°C for 20 min) and stored at -20 °C. Biochemical analyses were conducted on the obtained kidney samples which were stored in 10% natural buffer formalin. After tissue processing, the samples were blocked in cylindrical paraffin blockers and then stained by Hematoxilin-eosin (Dhandapani et al., 2002 ▶) and Periodic Acid Schiff [PAS] (each sample's diameter was 5-6 microns). The sections were examined microscopically for the evaluation of histopathological changes at 400× magnification.

**Statistical analysis**

All values are expressed as Mean±SEM. The differences were compared using the SPSS statistical analysis package (one-way ANOVA and Tukey test, p<0.05).

## Results

Table 1 shows the effect of hydro-alcoholic extracts of *R. ribes *root and glibenclamide on body weight and glucose content. The levels of glucose in serum of alloxan induced diabetic rats were significantly (p<0.05) elevated in comparison to the control group. Administration of* R. ribes *root (75 and150 mg/kg) or glibenclamide (0.6 mg/kg bw) to diabetic rats for 28 days caused significant reduction (p<0.05) in serum glucose level in comparison with diabetic control. Considerable reduction (p<0.05) also is found in the body weight of the diabetic rats comparing to control groups. Extract (75 and 150 mg/kg) or glibenclamide (0.6 mg/kg bw) treated groups showed an increase (p<0.05) in body weight in comparison to the diabetic control group. As shown in Table 2, alloxan caused significant hyperlipidemic action, where asignificant increase was recorded in the levels of LDL-cholesterol. Post-treatment with extracts of *R. ribes* root diabetic rats raised significant reduction in the LDL-cholesterol in comparison to the diabetic group, but HDL-cholesterol recorded significant elevation in comparing to the diabetic group. As it is shown in Table 2, the reduction in the levels of LDL-cholesterol reaches normal levels. Furthermore, in diabetic rats the levels of plasma HDL-cholesterol in pretreated group with *R. ribes* recorded a significant increase when compared with the normal group; in contrast, LDL-cholesterol recorded a significant decrease when compared with the control group (Table 2).

**Table 1:** The body weight and blood Glucose of Rats under treatments

**Groups**	** NC**		**N+RR(150 mg/kg)**	**DC**	**D+RR** **(75 mg/kg)**	**D+RR** **(150 mg/kg)**	**D+G** **(0.6 mg/kg)**
**Parameters**							
**FBG (mg/dl)**		104.16±3.28	98.44±6.83	371.66±6.83^b^	196.65±4.42^c^	118.67±1.43	111.27±3.63
**Body Weight (g)**		240.5±1.78	232.52±3.91	139.76±3.75^b^	187±1.53^c^	202.63±5.02^d^	206.55±3.46^d^

RR: *R. ribes *root, N: normal, C: control, G: glibenclamide, D: diabetic.Values are presented as mean±SEM; n=6 in each group. One way ANOVA followed by Tukey’s test. Similar words show no difference while un-similar words show significant difference (p<0.05).

**Table 2:** Some biochemical blood factors of Rats under treatments

**Groups**	** NC**		**N+RR** **(150 mg/kg)**	**DC**	**D+RR** **(75 mg/kg)**	**D+RR** **(150 mg/kg)**	**D+G** **(0.6 mg/kg)**
**parameters**							
**Serum Creatinine (mg/dl)**		0.78±0.02	0.79± 0.08^a^	1.72± 0.06^b^	1.30±0.04^c^	0.90±0.01^d^	0.77±0.06^a^
**Serum urea(mg/dl)**		22.11±0.72	21.71± .34	72.3±0.17^b^	39.33±2.38^c^	26.44±1.10	23.57±1.06
**Cholesterol(mg/dl)**		106.37±0.49	101.22±1.69	142.54±2.68^b^	114.66±.74^c^	105.66±2.59	118.81±2.204^c^
**Triglyceride(mg/dl**		62.29±.82	61.46±1.08	132.20±1.71^b^	87 ±2.09^c^	69.05±3.09	82.19±3.00^c^
**LDL.Ch.(mg/dl)**		25.46±0.67	26.02±08	60.96±4.32^b^	34.04±.07^c^	22.95±0.84	24.5±1.18
**HDL.Ch.(mg/dl)**		48.5±0.44	51.55±2	34.55±0.49^b^	43.3±1.16^c^	49 .55±49	47.79± 0.30

RR: *R. ribes* root, N: normal, C: control, G: glibenclamide, D: diabetic.Values are presented as mean±SEM; n=6 in each group. One way ANOVA followed by Tukey’s test. Similar words show no difference while un-similar words show significant difference (p<0.05).

The mean values of serum creatinine, serum urea, of both control and experimental groups, are presented in Table 2. Alloxan-induced diabetic rats showed a significant increase (p<0.05) in serum creatinine, while serum urea significantly increase (p<0.05) in comparison with the normal control. There was a significant restoration of these parameters to near normal levels after administration of the of *R. ribes *root and also by glibenclamide (0.6 mg/kg bw).

**Histopathology of kidney**

In diabetic rats, kidney sections showed mild thickening of the basement membrane along with mild change in the density of mesangial cells, atrophy of glomerular capillaries with increased Bowman's space (urinary space), and acute tubular necrosis (NT). The groups that were treated with *R. ribes *root (75 and 150 mg/kg) or glibenclamide (0.6 mg/kg bw) depicted features of healing, i.e., normal glomerulus, normal basement membrane, and capillaries. Moreover, Bowman's space (urinary space) and acute tubular necrosis (NT) were improved towards normal condition after treatment with *R. ribes *root (75 and 150 mg/kg) or glibenclamide (0.6 mg/kg bw).

**Figure1:**
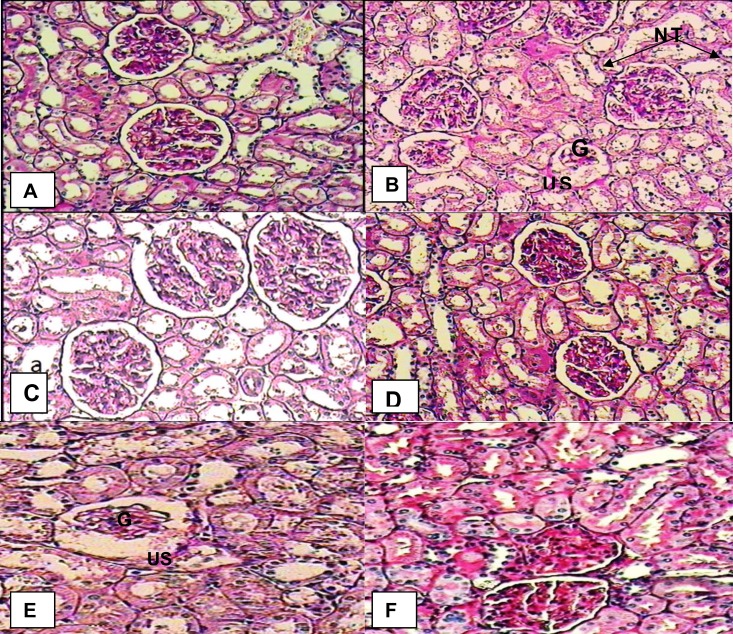
Histopathological evaluation of kidney sections. Various panels represent control kidney (A), diabetic kidney (B) atrophy of glomerulus capillaries (G), increased Bowman's space (urinary space) (US) and kg acute tubular necrosis (NT), diabetic kidney treated by 75 mg/kg of *R. ribes* root (C), diabetic kidney treated by 150 mg of *R. ribes* root( D), diabetic kidney treated by 0.6 mg/kg of glibenclamide (E) and Positive control treated withhydroalchlic extract *R. ribes *of root (150 mg/kg body weight) (F) with normal glomerulus

## Discussion

It is well understood that the specific toxicity of alloxan on Beta-cells of pancreas is due to quick absorption of alloxan by pancreatic Bata-cells and free radicals production by alloxan. Free radicals can cause reversible or irreversible damages to cellular compound (such as proteins, lipids, carbohydrates, nucleic acids, etc.) and thereby affect cellular activities such as function of membrane, metabolism, and gene expression which may cause some diseases such as atherosclerosis, cancer, mellitus diabetes, etc. (Szkudelski, 2001 ▶). Antioxidants also are able to absorb free radicals, which became inactive later by transferred electron from the membrane (Fukuda et al., 2004 ▶; Vaya and Aviram, 2002 ▶).

In present study, significant increase in serum glucose with monohydrate alloxan treatment can be attributed to distraction of Langerhans β-cells which is in good agreement of Byung-Hyun Park findings (Byung-Hyun and Jin-Woo, 2001 ▶). By increasing blood sugar content in diabetic rats following injecting alloxan, triglyceride content also increased which is similar to previous reports (Broadhurst, 1997 ▶). The observed increase in LDL and HDL content in diabetic rats can be found similar to previous reports (Winocour et al., 1992 ▶; Abou-Seif and Yussef, 2004 ▶).

We have observed that treatment of diabetic rats with Rhubarb, lead to sharp reduction in plasma glucose. This result can be associated with modification of glucose absorption and translocation by competition of aglycones sit due to the anti-neoplastic potentials of the main anthraquinones from Rhubarb (Fairbairn, 1977 ▶). This therapeutic effect of Rhubarb was presumably attributed to direct increasing in large intestinal tone which leads to decrease intestinal stealing of glucose and increase of excretion (Sim, 1967 ▶). Vaoler and their colleagues explained the glucose reduction in diabetic patient to fiber diet which reduce post parental blood sugar by forming viscous gel in contact with water which delay the absorption of carbohydrate in small intestine. Rhubarb contains 74% fiber, i.e. (66% insoluble and 8% soluble), which may lead to certain physiological activity (Vaoler et al., 1981 ▶).

It was also suggested that Rhubarb fiber increases pro-glucagon gene expression and modulates intestinal glucose uptake which is promoted the reduction of blood glucose (Reime et al., 2008 ▶). Goel et al reported the hypoglycemic effect of Rhubarb to epicatechin pharmacologically active substance of Rhubarb, contains (water-soluble tannin), which is most closely related to β-cell stimulation of pancreas. It was already reported that the epicatechin can promote β-cell activity according to the level of hyperglycemia (Goel et al, 1997 ▶). Rhubarb treatment (75 and 150) groups displayed improvement of body weight (Table 1) comparing to diabetic groups. These results of body weight improvement may be associated to positive modification of blood sugar, which improved weight gain through successful glucose utilization. (Fairbairn, 1977 ▶). In present study, the post-treatment of diabetic rats with Rhubarb extract lead to reduction of plasma cholesterol, triglycerides and LDL–cholesterol whereas plasma HDL-cholesterol level statistically increased. These findings are in agreement with previous studies; FalahHosseini et al showed that in R. ribes treated groups blood glucose, triglyceride, LDL and total cholesterol level at the end of the study were significantly decreased as compared to beginning of the study.

Li and Liu have reported that Rheum treatment in 5/6 nephrectomised rats can decrease significantly plasma cholesterol and triglycerides (Li and Liu, 1991 ▶). Li and Ye have also reported that the Rheum in normal and hyperlipidemia rabbits significantly reduced serum cholesterol, serum triglyceride/p-Lipoprotein (LDL) and pre-p lipoprotein (VLDL) (Li and Ye, 1981 ▶). This result also supports the findings of Goel et al, that daily ingestion of Rhubarb stalk fiber in hypercholesterolemia men for 4 weeks can significantly reduce serum total cholesterol and LDL-cholesterol, while the HDL-cholesterol level remained unchanged (Goel et al., 1997 ▶). Their results clearly demonstrate the potential effects of Rhubarb on cholesterol lowering in men. This report also reveals that cholesterol-supplemented rats, Rheum rhaponticum stalk fiber significantly reduced plasma cholesterol, hepatic concentration of cholesterol and cholesteryl esters content. Rhubarb fiber feeding significantly reduced the activity of acyl-coA and cholesterol acyl transferees; and also increased the fecal bile acid loss and the activity of cholesterol 70.-hydroxylase (Goel et al., 1997 ▶). Abe et al, have suggested that the cholesterol lowering effect of Rhubarb (R. palmatum) may be due to the potent inhibitory effect of squalene poxidase which is a rate-limiting enzyme of cholesterol biosynthesis (Abe et al., 2000 ▶). They proposed that the hypocholesterolemic effect of Rhubarb fiber may be due to the increased excretion of bile acid and induction hydroxylase activity in rats. These results are in agreement with our findings that we have observed the cholesterol lowering effect of RR which is another Rhubarb species.

The diabetic hyperglycemia induces the elevation of the plasma urea and creatinine in diabetic rats, which are considered a significant marker of renal dysfunction (Almdal and Vilstriup, 1988 ▶). Histopathological findings of kidney in diabetic rats showed atrophy of glomerular capillaries with acute tubular necrosis, while in treated groups with hydroalchlic extract *R. ribes* of root, this disorders could be protected. In these groups all of renal injury symptoms were improved towards normal condition.

In the present study the effect of Rhubarb on the kidney functions was assessed by the determination the levels of plasma creatinine and urea. According to previous reports post-administration of Rhubarb extract to the diabetic rats can decreased the level of plasma creatinine and urea (Li Leishi., 1996 ▶). Furthermore, the treatment of Rhubarb extract could significantly prevent depletion of antioxidant concentration andantioxidant enzymatic activities in the kidneys.

Additionally, the presence of polyphenols and flavonoids in Rhubarb extract might be responsible for the antioxidant nephron-protective activities and the reduction of serum urea and creatinine levels. According to literature Rhubarb is one of the most widely used plants in Chinese medicine and has been applied in clinic to treat kidney diseases for years. Recent experimental discoveries offer important evidence of rhubarb's effect on kidney failure. In astudy on diabetic rats with nephropathy, Rhubarb extract stopped the swelling (renal hypertrophy) at an early stage, and so might be useful in the early stages of human diabetic kidney disease (Yokozawa et al., 1991 ▶). Also a number of studies have observed the effects of Rhubarb in rats with CRF. Low molecular weight tannins, purified from Rhubarb, produced an increase in glomerular filtration rate, decreased levels of uremic toxins, and increased blood flow to the kidneys (Peng et al., 2005 ▶; Yarnell, 2002 ▶).

In conclusion and according to the results of biochemical and histopathological studies, Rhubarb can be used to decrease plasma Glucose and cholesterol levels (especially at dose of 150 mg/kg bw). It is concluded that post-treatment with Rhubarb extract cause a significant anti-hyperglycemic effect. It seems that Rhubarb is capable to improve hyperlipidemia which involves kidney functions in diabetic rats. However, it is not known whether reported lipid-lowering effects are associated with the improvement of endothelial function.

## References

[B1] Abe I, Seki T, Noguchi H (2000). Galloyl esters from rhubarb are potent inhibitors of squaleneopoxidase, a key in cholesterol biosynthesis. Planta Med.

[B2] Abou-Seif MA, Yussef AA (2004). Evaluation of some biochemical changes in diabetic patients. Clin Chem Acta.

[B3] Almdal TP, Vilstrup H (1988). Strict insulin treatment normalizes the organic nitrogen contents and the capacity of urea-N synthesis in experimental diabetes in rats. Diabetologica.

[B4] BaytopT (1999). Therapy with Medicinal Plants in Turkey.

[B5] Broadhurst CL (1997). Nutrition and non-insulin dependent diabetes from ananthropologicalperspective. Alt MedRev.

[B6] Budavari S (1989). The mesk index an encyclopedia of chemicals. Drug sbiological.

[B7] Byung-Hyun Park, Jin-Woo Park (2001). The protective effect of Amomumxanthides extracts against alloxan-induced diabetic rats through the suppression of NF Bactivation. Exp Med.

[B8] DCCT (2002). Writing Team for the Diabetes Control and Complications Trial/ Epidemiology of Diabetes Interventions and Complication Research Group, Effect of Intensive Therapy on the Microvascular Complications of type 1 Diabetes mellitus”. JAMA.

[B9] Dhandapani S, Subramanian V, Rajagopal S, Namasivayam N (2002). Hypolipidemic effect of CuminumcyminumL onalloxan– induced diabetic rats.. Phar Res.

[B10] Eddouks M, Maghrani M (2004). Phlorizin-like effect of Fraxinusexcelsiorin normal and diabetic rats. J Ethnopharmacol.

[B11] Fairbairn JW (1977). Anthraquin one laxatives.

[B12] Fukuda T, Ito H, Yoshida T (2004). Effect of the walnut polyphenol fraction on oxidative stress in type 2 diabetes mice. Biofactors.

[B13] FalahHosseini H, Heshmat R, Mohseni F, Jamshidi Ah, Alavi SHR, Ahvazi M, Ardeshir Larijani MB (2008). The Efficacy Of Rheum Ribes L. Stalk Extract On Lipid Profile In Hypercholesterolemic Type II Diabetic Patients: A Randomized, Double-Blind, Placebo-Controlled, Clinical Trial. J Med Plants.

[B14] Ghorbani A (2013). Best herbs for managing diabetes: A review of clinical studies. Braz J Pharm Sci.

[B15] Goel V, Ooraikol B, Basu TK (1997). Cholesterol lowering effects of Rhubarb stack fiber in hypercholesterolemic men. Journal of American College of Nutrition Dec.

[B16] Hadjzadeh MA, Parsaee H, Sadeghian A (2004). Cholesterol lowering effect of Rheum ribes in hypercholesterolemic rabbits. Med J Islam Repub Iran.

[B17] Halder N, Joshi S, Gupta SK (2003). Lens aldose reductase inhibiting potential of some indigenous plants. J Ethnopharmacol.

[B18] Huang YH, Shi M, Zheng WD, Zhang LJ, Chen ZX, Wang XZ (2006). Therapeutic effect of interleukin -10 on CC14-induced hepatic fibrosis in rats. World J Gastroenterol.

[B19] Jothivel N, Ponnusamy SP, Appachi M, Singaravel S, Rasilingam D, Deivasigamani K, Thangavel S (2007). Anti-diabetic activity of methanol leaf extract of Costuspictus D DON in alloxan-induced diabetic rats.. Health Sci.

[B20] Kazemi S, Asgary S, Moshtaghian J, Rafieian M, Adelnia A (2010). Liver-protective Between hyperfiltration and impairment: demystifying early renal functional changes in diabetic nephropathy. Diabetes Res Clin Pract.

[B21] Krishnaiah DR, Sabatly R, Nithyanandam R (2010). A review of the antioxidant potential of medicinal plant species. J Food Bioproducts Processing.

[B22] Li CL, Ye YW (1981). Effect of rhapontin of Rheum hotaoense on lipidand lipoprotein level serum. Acta Pharm Sinica.

[B23] Yang JW, Li LS (1993). Effect of Rheum on renal hypertrophy and hyper filtration of experimental diabetes in rat. Zhongguo Zhong Xi Yi Jie He Za Zhi.

[B24] Li Leishi (1996). End Stage renal disease in China. Kidney International.

[B25] Li LS, Liu ZH (1991). Clinical and experimental studies of rheum on preventing progression of chronic renal failure. Zhong Xi Yi Jie He Za Zhi.

[B26] Liu CT, Sheen LY, Lii CK (2007). Does garlic have a role as an antidiabetic agent? Mol. Nutr. Food Res.

[B27] Lokesh D, Amit SD (2006). Diabetes mellitus- its possible pharmacological evaluation technique sand naturotherapy. Int J Green Pharm.

[B28] Machlean W, Townsend P (2001). Rhubarb (Dahung). Rheum palmatum.

[B29] Malviya N, Jain S, Malviya S (2010). Antidiabetic Potential of Medicinal Plants. Acta Pol Pharm.

[B30] Marles RJ, Farnsworth N (1996). Antidiabetic Plants and their Active Constituents: An update. Prot J Bot Med.

[B31] Matsuda H, Morikawa T, Toguchida I, Park J Y, Harima S, Yoshikawa M (2001). Antioxidant constituents from rhubarb: Structural requirements of stilbenes for the activity and structures of two new anthraquinoneglucosides. Bioorg Med Chem.

[B32] Ozbek H, Ceylan E, Kara M, Ozgokce F, Koyuncu M (2004). Hypoglycemic effect of Rheum ribesroots in alloxan induced diabetic and normal mice. Scand J Lab Anim Sci.

[B33] Peng A, Gu Y, Lin SY (2005). Herbal treatment for renal diseases. Ann Acad Med Singapore.

[B34] Ranjan C, Ramanujam R (2002). Diabetes and insulin resistance associateddisorders: Disease and the therapy. Curr Sci.

[B35] Reimer RA, Russell JC (2008). Glucose tolerance, lipids and GLP-1secretion in JCR: LA-cp rats fed a high protein fiber diet. Obesity.

[B36] Shaw JE, Sicree RA, Zimmet PZ (2010). Global Estimates of the Prevalence of Diabetes for 2010 and 2030. Diabetes Res Clin Pract.

[B37] Sim Sk (1967). Medplant glycoside.

[B38] Szkudelski T (2001). The mechanism of Alloxan and Streptozotocin action in B cells of the rat pancreas. Physiol Res.

[B39] Tuzlacı E, Meriçli AH (1991). Some studies on Isgın (Rheum ribes) and its distribution in Turkey.

[B40] Tyler VE, Brady LR, Robbers JE (1988). Pharmacognosy.

[B41] Vaoler S, Hassen KF, Aogenaes Q (1981). Effects of different kinds of fiber on post prandial blood glucose in insulin dependent diabetes mellitus. Acta Med Scanda.

[B42] Vaya J, Aviram M (2002). Nutritional antioxidants: mechanism of action, analyses of activities and medical applications. Curr Med Chem Immunol Endocr Metab Agents.

[B43] Winocour PH, Durrington PN, Bhatnagar D, Ishola M, Arrol S, Mackness M (1992). Abnormalities of VLDL, IDL and LDL characterize insulin dependent diabetes mellitus. Arteroscler Thromb.

[B44] Yarnell E (2002). Botanical medicines for the urinary tract. World J Urol.

[B45] Yokozawa T, Fujioka K, Oura H, Nonaka G, Nishioka I (1991). Effects of rhubarb tannins on uremic toxins. Nephron.

